# Involvement of lncRNAs and Macrophages: Potential Regulatory Link to Angiogenesis

**DOI:** 10.1155/2020/1704631

**Published:** 2020-02-29

**Authors:** Yang Jia, Yedi Zhou

**Affiliations:** ^1^Department of Pediatrics, The Second Xiangya Hospital, Central South University, Changsha, Hunan 410011, China; ^2^Department of Ophthalmology, The Second Xiangya Hospital, Central South University, Changsha, Hunan 410011, China; ^3^Hunan Clinical Research Center of Ophthalmic Disease, Changsha, Hunan 410011, China

## Abstract

Macrophages are involved in angiogenesis, an essential process for organ growth and tissue repair, and could contribute to the pathogenesis of angiogenesis-related diseases such as malignant tumors and diabetic retinopathy. Recently, long noncoding RNAs (lncRNAs) have been proved to be important in cell differentiation, organismal development, and various diseases of pathological angiogenesis. Moreover, it has been indicated that numerous lncRNAs exhibit different functions in macrophage infiltration and polarization and regulate the secretion of inflammatory cytokines released by macrophages. Therefore, the focus of macrophage-related lncRNAs could be considered to be a potential method in therapeutic targeting angiogenesis-related diseases. This review mainly summarizes the roles played by lncRNAs which associated with macrophages in angiogenesis. The possible mechanisms of the regulatory link between lncRNAs and macrophages in various angiogenesis-related diseases were also discussed.

## 1. Introduction

Angiogenesis is the growth process of blood vessels and plays important roles in the physiological functions for organ growth and tissue repair [1], as well as a large number of angiogenesis-related diseases such as tumors, arthritis, diabetic retinopathy, and age-related macular degeneration [2]. Targeting angiogenesis is an effective therapeutic method for anticancer treatment and has been applied in many kinds of cancer (e.g., lung cancer [3, 4] and gastric cancer [5]). The treatment of antivascular endothelial growth factor (VEGF) has been applied in inhibiting angiogenesis, especially in cancer [6] and ocular diseases [7]. However, beyond VEGF, there are also a variety of other molecules that play important roles in the mechanisms of angiogenesis [8, 9].

Long noncoding RNAs (lncRNAs) are those which demonstrate no apparent protein-coding capacity and longer than 200 nucleotides [10]. Recent studies indicated a variety of regulatory functions of lncRNAs in a wide range of cellular and developmental processes as well as pathogenesis [11–15]. In particular, lncRNAs control cell differentiation and self-renewal through neural, skin, and muscle stem cells [16]. LncRNAs are also involved in diseases of pathological angiogenesis, such as diabetic retinopathy [17, 18].

Macrophages are important angiogenic effector cells and act as key modulators in both tumor growth and angiogenesis [19]. Many studies suggested that under various stimuli, macrophages could be polarized to two phenotypes: classically activated M1 phenotype and alternatively activated M2 phenotype [20–22]. Those M1 macrophages can destroy foreign organisms and inhibit tumor growth, while M2 phenotype functions in wound healing, chronic infections, tumor growth, and angiogenesis [23–29]. We previously revealed that M2 macrophages, rather than M1 phenotype, infiltrated in the inner layer of the retinas of oxygen-induced retinopathy and enhanced retinal neovascularization *in vivo* [30]. In a choroidal neovascularization mouse model, we recognized that M1 and M2 macrophages have different distributions, thus might have diverse potential biological functions in angiogenesis [31]. A recent study reported that lncRNA MM2P regulated tumorigenesis and angiogenesis via modulating M2-like macrophage polarization [32], indicating that lncRNAs and macrophages might be involved and have a potential regulatory link to angiogenesis.

In the present review, we summarize the roles of lncRNAs associated with macrophages in angiogenesis and discuss the possible mechanisms of the regulatory link between lncRNAs and macrophages in various angiogenesis-related diseases.

## 2. LncRNAs Regulate Macrophage Infiltration, Polarization, and Functions

Monocytes are considered as the precursors of macrophages, originated from hematopoietic stem cells, and monocyte/macrophage differentiation plays a critical role in response to the immune system and pathological diseases [33–35]. It has been indicated that lncRNA lnc-MC was involved in monocyte/macrophage differentiation, positively regulated by PU.1, a hematopoiesis-specific transcription factor, and negatively interacted with miR-199a to promote differentiation process [36]. Besides monocyte/macrophage differentiation, lncRNAs seemed to be involved in macrophage infiltration. For example, downregulation of LRNA9884 significantly suppressed macrophage infiltration by reducing the level of monocyte chemoattractant protein-1 (MCP-1) in a type 2 diabetic nephropathy mice model [37]. Moreover, lncRNA CASC2c could inhibit macrophage migration and M2 polarization by negatively regulating the expression of coagulation factor X, which was reported to promote the infiltration of macrophages to the glioblastoma multiforme tumor cells, and polarize macrophages to M2 phenotype [38]. In contrast, activated lncRNA UCA1 promoted macrophage infiltration, resulting in carcinogenesis and progression of breast cancer [39].

LncRNAs could also induce macrophage polarization and lead to regulatory effects on their functions. Lipopolysaccharide (LPS) and interleukin- (IL-) 4 induction was commonly applied for M1/M2 macrophage polarization, respectively [32]. Ye et al. observed that lncRNA Cox-2 is expressed higher in LPS-induced M1 macrophages than IL-4-induced M2 macrophages, and silencing lncRNA Cox-2 expression markedly altered the macrophage polarization from M1 to M2 phenotype [40]. In addition, lncRNA Cox-2 siRNA significantly enhanced the ability of macrophages in tumor proliferation, invasion, and migration by mediating M1/M2 polarization [40]. Moreover, using the gain-of-function and loss-of-function strategies, lncRNA TUC339 was recognized to be required for macrophage polarization to regulate the release of pro- or anti-inflammatory cytokines and thereby affect tumor growth [41]. Overexpression of TUC339 in hepatocellular carcinoma (HCC) cells suppressed the expression of proinflammatory factors, such as IL-1*β* and TNF-*α*, and knockdown of TUC339 obtained an opposite effect [41]. It has been reported that LPS could strengthen the lncRNA CCL2 levels to mediate the expressions of inflammatory factors in macrophages, and this enhancement could be suppressed by SIRT1 in sepsis [42]. Knockdown of lncRNA CCL2 resulted in a reduction of IL-1*β*, IL-6, and TNF-*α* [42]. In response to LPS, lncRNA Nfkb2 and lncRNA Rel, located near proinflammatory transcription genes, were increased and closely related to the inflammatory response in mouse macrophages [43].

Some lncRNAs could target related molecules or signaling pathways to regulate macrophage polarization. For example, lncRNA GAS5 was significantly reduced in M2-polarized microglia, and overexpression of GAS5 suppressed microglial M2 polarization via inhibition of transcription of IRF4, which is an important regulatory molecule of M2 polarization [44]. As we discussed, lncRNA MM2P was higher expressed in M2 macrophages rather than in M1 macrophages, and blockade of lncRNA MM2P could weaken the IL-4/STAT6 signaling pathway, resulting in a reduction of both cytokine-regulated M2 polarization and M2-induced angiogenesis [32]. NF-*κ*B, which is a downstream signaling pathway of toll-like receptors (TLRs) after specific microbial and pathogen recognition, could induce transcription of proinflammatory genes and is strongly involved in the regulation of macrophage polarization [45]. After LPS stimulation, the expression of lncRNA Mirt2 was induced in macrophages and suppressed the proinflammatory factors (such as TNF, IL-1*β*, IL-6, and IL-12) by inhibiting the activation of NF-*κ*B and MAPK pathways [46]. In contrast to LPS, Mirt2 also could promote the polarization of M2 macrophages induced by IL-4, but the mechanism might be independent from STAT6 and PPAR*γ* pathways [46]. Under LPS-mediated inflammatory conditions, lncRNA Tnfaip3 exerts a coregulatory role with NF-*κ*B in modulating inflammatory gene transcription in macrophages [47]. Another NF-*κ*B-mediated lncRNA FIRRE exhibited posttranscriptional elevation of inflammatory genes in macrophages and epithelial cells by interacting with heterogeneous nuclear ribonucleoproteins U after LPS stimulating [48]. Overall, these studies showed that the expression profiles of lncRNAs can be clearly distinguished between M1 and M2 macrophages, indicating that lncRNAs could be involved in regulating macrophage polarization. Dysregulation of lncRNAs may affect macrophage polarization by targeting both downstream signaling pathways and the release of inflammation cytokines.

According to competing endogenous RNA (ceRNA) networks, lncRNAs could act as sponges to regulate the functions of miRNAs [49]. Studies had demonstrated that lncRNA NIFK-AS1 and lncRNA CCAT1 could inhibit the polarization of M2 macrophages by targeting miR146a and miR-148a, respectively [50, 51]. Moreover, lncRNA XIST and lncRNA GNAS-AS1 exhibited the promotion of M2 polarization, such functions were associated with T-cell-specific transcription factor 4 (TCF-4) and miR-4319, respectively [52, 53].

MALAT1 is an important lncRNA that has been widely investigated [18, 54–57]. Recent studies had reported that the MALAT1 regulates the production of inflammatory cytokines [56] and was increased in a LPS-induced acute lung injury model to regulate the release of IL-1*β*, IL-6, and TNF-*α* [58]. Silencing of MALAT1 inhibited the proinflammatory responses by enhancing miR-146a levels in macrophages and epithelial cells [58]. In LPS-induced septic cardiomyocytes, expression of MALAT1 was induced by IL-6 and elevated the production of TNF-*α* partially through serum amyloid antigen 3 (SAA3) [59]. By targeting SAA3, MALAT1 also could modulate the expression of IL-6 and TNF-*α* in the endothelial cells under high-glucose conditions [60]. Although the proinflammatory activities of MALAT1 in macrophages were reported, Zhao et al. presented an opposite effect of MALAT1, which functions as an anti-inflammatory regulation *in vitro* [61]. In this study, scientists had demonstrated that MALAT1 was upregulated by LPS to suppress the production of proinflammatory TNF-*α* and IL-6 by interacting with the NF-*κ*B pathway in macrophages. The knockdown of MALAT1 achieved enhancement of TNF-*α* and IL-6 [61]. It is known that tumor-associated macrophages (TAMs) exhibit similar functions to M2 macrophages [62] and MALAT1 was upregulated in TAMs compared to nonpolarized macrophages and promoted angiogenesis through secretion of fibroblast growth factor-2 (FGF2) protein [63]. Moreover, in macrophages, MALAT1 regulates lysosomal-associated membrane protein 1 (lamp1) expression by sponging miR-23-3p [64]. For the above contradictory effect of MALAT1 in inflammatory responses, further investigations are required to reveal the essential mechanisms of MALAT1 to macrophage functions in angiogenesis.

**Figure 1:**
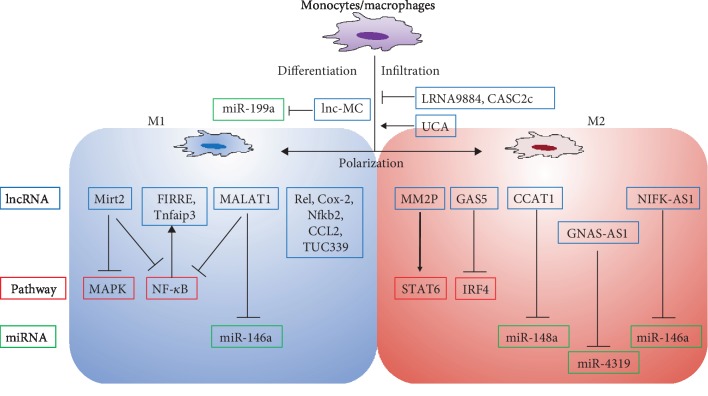
The mechanisms of lncRNAs regulate macrophage infiltration, polarization, and functions.

Together, the above studies suggest that lncRNAs could regulate macrophage infiltration, polarization, inflammation, and secretion by targeting various pathways to change the pro- and/or anti-inflammatory response mechanisms (Figure 1). Further studies on the mechanism of lncRNAs in macrophages can lead to enhance the understanding on how lncRNAs might be involved in inflammation and thereby affect the regulation of immune response of angiogenesis.

## 3. Link between Macrophages and Angiogenesis

It is widely considered that M1 macrophages present a proinflammatory effect and M2 macrophages present an anti-inflammatory effect. Besides, M2 macrophages also induce proangiogenic functions, and the induction of M2 macrophages enhances cancer invasion and metastasis, as well as the development of neovascular diseases through VEGF [65–67]. By now, although the activation of the downstream pathway during angiogenesis is still not completely clear, activated macrophages could influence the angiogenic process through the production of angiogenic factors such as IL-1, IL-6, IL-8, TNF-*α*, TGF-*α*, TGF-*β*, GM-CSF, bFGF, and VEGF [68]. Moreover, activation of NF-*κ*B and STAT3 is involved in the upstream pathway of macrophage-induced angiogenesis [69, 70]. Thus, macrophages and angiogenesis are very closely linked with complicated mechanisms.

## 4. Involvement of lncRNAs in the Pathogenesis of Angiogenesis-Related Diseases

### 4.1. Tumor Angiogenesis

Many studies revealed the involvement of lncRNAs in the recruitment of macrophages to tumor cells and M1/M2 polarization of macrophages to change the tumor microenvironment.

As we discussed before, MALAT1 not only acted as a potential cancer biomarker [54] but also regulated angiogenesis in diabetic retinopathy [71], tumor [63, 72–74], hindlimb ischemia [75], and brain vascular endothelium [76]. In particular, as we described, Huang et al. reported that MALAT1 enhanced thyroid cancer angiogenesis by regulating FGF2 secretion of TAMs [63]. In HCC cells, MALAT1 could promote angiogenesis and regulate polarization of macrophages through sponging miR-140 [74]. These suggested that macrophages might be an important modulator of angiogenesis in the mechanisms of MALAT1.

As mentioned above, MM2P could contribute to promoting M2 polarization of macrophages and inducing angiogenesis, resulting in tumor deterioration [32]. As we described, lncRNA UCA1 was demonstrated to be involved in macrophage recruitment to promote breast cancer invasion in a previous study [39]. In cervical cancer cells, UCA1 was upexpressed and negatively associated with miR-206, and knockdown of UCA1 directly decreased VEGF through miR-206 upregulation, and thereby suppressed tumor growth, viability, migration, and invasion [77]. Another lncRNA TUC339 was significantly increased in cancer stem cell-derived exosomes, and VEGF was enhanced in exosomes derived from cancer stem cells correspondingly [78]. The knockdown of TUC339 reduced HCC cell growth and spread [79]. The mechanism has been uncovered that TUC339 could regulate the macrophage polarization, functioning as promotion of anti-inflammatory cytokines and angiogenesis, thereby accelerating tumor proliferation [41]. Sang et al. showed that lncRNA CamK-A was involved in macrophage infiltration and angiogenesis by triggering the transcription of the NF-*κ*B signaling pathway in tumor cells [80]. By promoting NF-*κ*B downstream cytokines (e.g., VEGF, IL-6, and TNF-*α*), Camk-A could remodel tumor microenvironment to recruit macrophages to tumors and contribute to angiogenesis [80]. LncRNA LNMAT1 upregulated CCL2 and recruited M2 macrophages to the tumor, and promoted lymphatic metastasis via excretion of VEGF-C [81]. These studies indicated that lncRNAs could regulate the recruitment of macrophages to the tumor, macrophage polarization, secretion of VEGF, and thereby the induction of pathological angiogenesis and tumor growth and spread.

It has been demonstrated that lncRNA PVT1 is involved in the high microvessel density in gastric cancer as well as the promotion of tumor growth through activation of the STAT3 signaling pathway as well as secretion of VEGFA [82]. The knockdown of lncRNA ROR was reported to reduce angiogenesis through inhibition of NF-*κ*B and JAK1/STAT3 pathways [83]. Moreover, overexpression of miR-26 could rescue the negative effects of ROR silencing, demonstrating that ROR functions as a molecular sponge for miR-26 in these activations [83]. LncRNA LIMT was suppressed by epidermal growth factor (EGF) and downregulated in breast cancer and ovarian cancer, and the EGF secreted from TAMs suppressed the levels of LIMT through activation of the EGF-ERK pathway [84, 85]. Although the direct links between these lncRNAs and macrophages were poorly indicated, it is possible that lncRNAs could interact with macrophage-related signaling pathways to regulate the tumor angiogenesis.

### 4.2. Angiogenesis in Other Diseases

Many major causes for blindness, such as age-related macular degeneration, retinopathy of prematurity, diabetic retinopathy, and retinal vein occlusions, are due to the pathological angiogenesis [86]. In particular, diabetic retinopathy, a complication of diabetes mellitus, is a major cause of blindness worldwide in which pathological processes are characterized by the formation of abnormal blood vessels within the eye [87]. LncRNAs could target macrophage-related signaling pathways to regulate the pathological angiogenesis. With the high-glucose treatment in human retinal endothelial cells, the expression of lncRNA ANRIL was increased and regulated VEGF expression through polycomb repressive complex 2 (PRC2) complex [88]. By binding to the NF-*κ*B signaling pathway, ANRIL could induce pathologic damage of retinopathy in the diabetic rat model [89]. Moreover, ANRIL could also promote angiogenesis by activating the NF-*κ*B pathway in diabetes combined with cerebral infraction in a rat model [90]. Similarly, the expression of lncRNA MIAT was also elevated on high glucose stress through impacting the VEGF signaling pathway, while knockdown of MIAT attenuated retinal vessel dysfunction [91]. Clinical investigations in diabetes patients had shown that increased expression of MIAT was markedly associated with diabetic retinopathy process, and the increased MIAT decreased the viability of ARPE-19 cells in vitro via targeting the TGF-*β*1 pathway [92]. The high-glucose conditions suppress the expression of lncRNA MEG3, whereas the rescue of MEG3 could delay diabetic retinopathy by inhibiting TGF-1 and VEGF levels [93]. In addition, MEG3 could also be regulated by activation of the PI3k/Akt pathway in diabetes mellitus-related microvascular dysfunction [94].

LncRNA NEAT1 was reported to be involved in M2 macrophage polarization [95] and could promote inflammation in macrophages [96, 97]. NEAT1 could accelerate angiogenesis by enhancing VEGF, SIRT1, and BCL-XL in brain microvascular endothelial cells [98]. Indeed, loss of NEAT1 expression exhibits downregulation of VEGF and upregulation of miR-377 resulting in antiangiogenesis and proapoptosis [98], while the mechanisms of macrophage polarization and functions lack investigation. In contrast, lncRNA MEG3 negatively regulated angiogenesis after ischemic stroke via suppressing the Notch pathway [99], and the silencing of MEG3 resulted in a proangiogenesis effect in vascular endothelial cells [100]. Yan et al. found MEG3 could be activated and participated in apoptosis of macrophages under oxidized low-density lipoprotein stimulation, indicating a novel role of MEG3/miR-204/CDKN2A pathway in macrophages [101]. Therefore, these two lncRNAs were reported to be related to both angiogenesis and macrophages in each study, and it is highly hypothesized that lncRNAs might alter macrophage functions to regulate pathological angiogenesis. On the other hand, we demonstrated that M2 macrophages, rather than M1, have essential functions in promoting retinal pathological neovasculization, while more experimental evidence is needed to support this hypothesis [30]. In our previous study, 198 upregulated and 175 downregulated lncRNAs were identified by microarray analysis in an oxygen-induced retinopathy mouse model [102]. Among them, we highlighted four validated lncRNAs that could be potentially involved in cell adhesion molecules and thereby affect the progress of pathological retinal angiogenesis [102]. In a mouse model of choroidal neovascularization induced by laser photocoagulation, we identified 716 altered lncRNAs, and the altered target genes of 7 validated lncRNAs were enriched in the immune system process and the chemokine signaling pathway [103]. Therefore, macrophages might also be involved in the immunological regulation associated with those altered lncRNAs.

Moreover, lncRNA could be involved in monocyte/macrophage differentiation to regulate the pathogenesis. For example, lncRNA NTT was reported to be elevated in rheumatoid arthritis and its activation contributes to monocyte/macrophage differentiation, resulting in the pathological process of rheumatoid arthritis [104].

**Figure 2:**
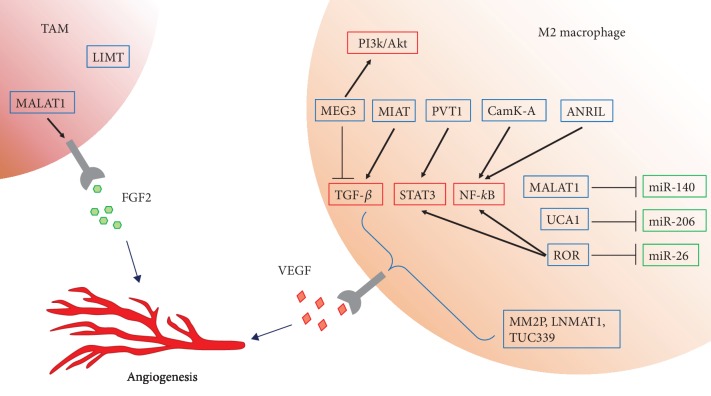
lncRNAs target macrophage-related pathways or miRNAs in macrophages to induce angiogenesis via elevating expression of VEGF or FGF2 in angiogenesis-related diseases.

Thus, lncRNAs are involved in various diseases associated with angiogenesis (Figure 2) and partially via the regulation of the functions of macrophages.

## 5. Summary

In sum, lncRNAs have been proved to play essential roles in angiogenesis in a variety of diseases. As shown in Figure 2, the mechanisms of direct effect to endothelial cells include regulating the secretion of growth factors or cytokines, such as VEGF or FGF2, and through a diverse range of pathways. On the other hand, some lncRNAs may also be associated with macrophage infiltration, differentiation, and polarization, and both lncRNAs and macrophages were involved in and have potential links to angiogenesis. Though some progress has been achieved in characterizing the functional lncRNAs in regulation of macrophage polarization, the mechanisms remain unclear, and further investigations are needed to understand the exact roles of lncRNAs which link to macrophages and angiogenesis. Therefore, targeting lncRNAs and the links with macrophages could be considered a novel therapeutic method in treating angiogenesis in different diseases.
